# Supplementary material for: Identification and characterization of intermediate states in mammalian neural crest cell epithelial to mesenchymal transition and delamination
Source: eLife. 2024 Jun 14;13:RP92844. doi: 10.7554/eLife.92844 (PMC11178358; doi:10.7554/eLife.92844)
Supplement: Supplementary file 1. [file elife-92844-supp1.docx]

Supplementary Table 1. NCC development related genes.

| **Gene** | **Expression pattern in relation to NCC development** | **References** |
| --- | --- | --- |
| ***Sox2*** | Neural epithelium (reduced in neural plate border) | Hafemeister & Satija, 2019; Lee et al., 2013; Wood & Episkopou, 1999 |
| ***Sox1*** | Neural epithelium (reduced in neural plate border) | Hafemeister & Satija, 2019; Lee et al., 2013; Wood & Episkopou, 1999 |
| ***Zic1/2*** | Neuroepithelium | Sauka-Spengler & Bronner-Fraser, 2008 |
| ***Wnt1*** | Neural plate border | Echelard et al., 1994; Parr et al., 1993 |
| ***Pax7*** | Neural plate border | Murdoch et al., 2012 |
| ***GCNF (Nr6a1)*** | Neural epithelium and early MNCC |  |
| ***Zeb2*** | Neural epithelium and early MNCC | Van de Putte et al., 2003 |
| ***Pax3*** | Neural plate border and early MNCC | Li et al., 2000 |
| **(Wnt1-Cre) EYFP** | Neural plate border and early MNCC | Hari et al., 2012 |
| ***Sox9*** | Neural plate border and early MNCC | Lee et al., 2013 |
| ***Foxd3*** | Neural plate border and early MNCC | Dottori et al., 2001 |
| ***Snail1*** | Neural plate border and early MNCC | Cheung et al., 2005 |
| **(Mef2c-F10N) LacZ** | Predominantly MNCC | Aoto et al., 2015 |
| ***Vimentin*** | MNCC (marker of mesenchymal cells) | Kobayashi et al., 2020 |
| ***Sox10*** | MNCC | Hari et al., 2012 |
| ***Twist1*** | MNCC | Soo et al., 2002 |
