# Supplementary material for: Identification and characterization of intermediate states in mammalian neural crest cell epithelial to mesenchymal transition and delamination
Source: eLife. 2024 Jun 14;13:RP92844. doi: 10.7554/eLife.92844 (PMC11178358; doi:10.7554/eLife.92844)
Supplement: Supplementary file 2. [file elife-92844-supp2.docx]

Supplementary Table 2. Primers for qRT-PCR

|  | Forward (5'-3') | Reverse (5'-3') |
| --- | --- | --- |
| *Dlc1* | AGCGGCTGTGAAAGAAA | GCATTACCCTTGGAGAAGA |
| *B2M* | CACTGACCGGCCTGTATGC | GGTGGCGTGAGTATACTTGAATTTG |
| *CANX* | CCAGACCCTGATGCAGAGAAG | CCTCCCATTCTCCGTCCATA |
